# The Effect of Corneal Irregularity on Astigmatism Measurement by Automated versus Ray Tracing Keratometry

**DOI:** 10.1097/MD.0000000000000677

**Published:** 2015-04-03

**Authors:** Hyun Cheol Roh, Roy S. Chuck, Jimmy K. Lee, Choul Yong Park

**Affiliations:** From the Department of Ophthalmology, Dongguk University, Ilsan Hospital, Goyang, Kyunggido, South Korea (HCR, CYP); and Department of Ophthalmology and Visual Sciences, Montefiore Medical Center, Albert Einstein College of Medicine, Bronx, NY, USA (RSC, JKL).

## Abstract

The aim of this study was to compare the effect of corneal irregularity on astigmatism assessment using automated keratometry (AK) (IOLMaster) versus ray tracing keratometry (Pentacam).

This is an observational case series approved by the institutional review board of Dongguk University Hospital, Goyang, South Korea.

A total of 207 eyes of 207 cataract patients were included. Preoperative corneal astigmatism was measured by both IOLMaster and Pentacam. Corneal irregularity index (IR) was calculated in Fourier analysis map of Pentacam. AK by IOLMaster and total corneal refractive power (TCRP, 3 mm and 4 mm zone analysis with pupil centered) by Pentacam were selected and the difference between the 2 measurements (delta Δ) was calculated using vector analysis. Ocular residual astigmatism (ORA) after cataract surgery was calculated by subtracting 6-month postoperative refractive astigmatism (RA) measurements from corresponding preoperative values (AK, TCRP3, and TCRP4).

The mean irregularity index measured was 0.042 ± 0.019 mm (mean ± standard deviation) and was positively correlated with age and magnitude of corneal astigmatism (*P* < 0.001 and *P* < 0.05). The difference (Δ) between TCRPs and AK (ΔTCRPs-AK) was 0.43 ± 0.37 (TCRP3) and 0.39 ± 0.35 (TCRP4) diopters. Linear regression analysis revealed that age (*P* < 0.001), IR (*P* < 0.001), and AK (*P* < 0.001) were positively correlated with ΔTCRPs-AK. In highly irregular corneas (IR over 0.77 diopters: mean + 2 standard deviation), postoperative ORAs calculated using TCRPs were significantly lower than ORAs calculated using AK.

Corneal irregularities significantly impact astigmatism assessment by IOLMaster (AK) and Pentacam (TCRPs). Compared with AK, TCRPs were more accurate in predicting postoperative residual astigmatism in highly irregular corneas.

## INTRODUCTION

The cornea is the most powerful refractive component of the human optical system. It is a prolate ellipse, with progressive flattening toward the periphery.^1^ Corneal shape and power were first measured using keratoscopic discs by Placido in 1880.^1^ Since then, numerous methods have been developed to measure corneal curvature, including manual and automated keratometry (AK), computer-assisted videokeratography, rotating Scheimpflug imaging, and optical coherence tomography (OCT).^2,3–9^

Curvature assessment of healthy corneas is highly reproducible whether by conventional keratometry or by advanced technology such as rotating Scheimpflug imaging or OCT.^10^ However, analyzing highly irregular corneas is challenging and cannot be accurately measured by conventional technology. Therefore, tomography-based corneal topography such as Scheimpflug imaging or OCT may be a better option to assess irregular corneas.^6^

Better interpretation of irregular corneas has become paramount in modern cataract surgery. Various techniques to minimize residual postoperative astigmatism are now available including, but not limited to, limbal relaxing incisions and toric intraocular lens (IOL). Minimizing residual astigmatism generally result in better visual acuity. However, applying these techniques to a highly irregular cornea is very challenging and often leads to unexpected refractive surprise.

Currently, it is not routine for cataract surgery candidates to receive preoperative topographic evaluation using Scheimpflug imaging or OCT. However, there are some patients who should be screened for corneal irregularity, including the elderly, where irregularity is more common.^11^

In this study, we compared 2 different keratometric strategies: AK from IOLMaster and total corneal refractive power (TCRP) from rotating Scheimpflug imaging device (Pentacam). We investigated which factors affect the discrepancy between corneal astigmatism measurement by AK and TCRP. In addition, by comparing the preoperative and postoperative astigmatism measurements, we determined which keratometric strategy, AK or TCRP, is better suited for astigmatism assessment in highly irregular corneas.

## MATERIALS AND METHODS

This study followed the tenets of the Declaration of Helsinki and was approved by the institutional review board of Dongguk University, Ilsan Hospital.

### Patients

A retrospective review of 435 eyes that underwent cataract surgery from May 2011 to May 2013 was performed and 207 right eyes of 207 patients (84 males and 123 females) were included in this study. Exclusion criteria included left eyes, existence of pterygium, history of previous corneal surgery, scars detected by slit lamp microscopy, poor data acquisition on rotating Scheimpflug imaging, eyes with any missing data, intraoperative complications including posterior capsule tears and zonule lysis, and early development of posterior capsule opacity before 6 months postoperatively.

### Preoperative Astigmatism Measurement

**FIGURE 1:**
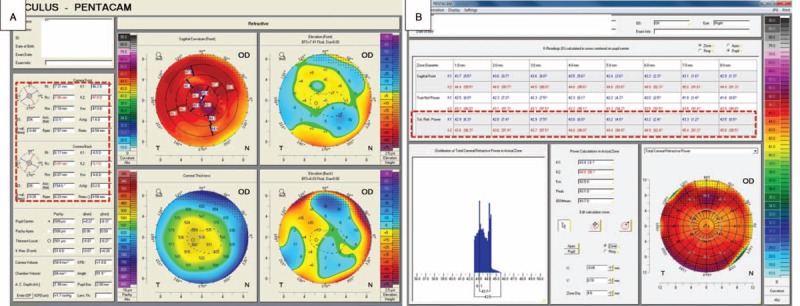
The screenshot of measurement in Pentacam. (A) The four refractive map. This map shows the anterior and posterior curvature and posterior astigmatism (red dotted area). Sagittal power, front and back elevation, and pachymetry were demonstrated in 4 separate graphics. (B) The screenshot for measurement of total corneal refractive power (TCRP). Several pupil centered zones with TCRP measurement are shown (red dotted area).

Preoperative corneal astigmatism was measured using IOLMaster (Carl Zeiss Meditec, Jena, Germany) and rotating Scheimpflug imaging (Pentacam® ver. 1.17r24; Oculus, Wetzlar, Germany). All measurements were performed in triplicate, and mean values were used for analysis. Among several keratometric values available in Pentacam, TCRP was selected and used for the analysis. Detailed information about TCRP is available at http://www.pentacam.com/sites/calc_corneal_power.php#ptop. TCRP is calculated by ray tracing, using a 3 (TCRP3) or 4-mm (TCRP4) diameter zone with the pupil center (Figure 1). AK from IOLMaster was used for analysis. Corneal irregularity index (IR) was automatically calculated in mm scale in Fourier analysis map, whereas total corneal irregular astigmatism at 4-mm zone is automatically calculated in μm scale in Cataract Pre-Op map of Pentacam. Mean anterior and posterior cornea curvature radii were measured by Pentacam at 3-mm central zone. And anterior to posterior curvature ratio was calculated. In the following, AK and TCRPs will be defined as the astigmatism obtained by their respective modalities when not otherwise specified.

### Vector Analysis

Each astigmatic value is represented by vectors Ã (C × A, where C is the positive cylinder value and A is the flat meridian) and then expressed using vector notation (Jackson coefficients; J0 = (C/2) × cos (2A), J45 = (C/2) × sin (2A). The J0 component represents the astigmatism with axes of 0 or 90 degree and the J45 component represents the astigmatism vector with axes of 45 or 135 degree.

The difference (Δ) between TCRP and AK (Δ TCRP-AK) between corneal astigmatisms was calculated using vectors:

 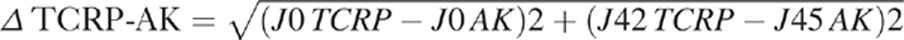


### Surgical Technique

Cataract surgery was performed using temporal clear corneal incisions (2.75 mm). A nontoric aspheric IOL (Tecnis^TM^, Abbot Medical Optics, Irvine, CA, USA) was inserted into the capsular bag after an IOL power was selected based on the SRK/T formula in IOLMaster. Postoperative regimen was as follows: topical 1% prednisolone and 0.5% levofloxacin applied 1 drop 4 times per day for 4 weeks, oral prednisone (10 mg) once per day for 7 days, and cefuroxime (500 mg) 2 times per day for 5 days. In diabetic patients, oral prednisone was omitted.

### Ocular Residual Astigmatism Measurement

Owing to the retrospective nature of this study, ocular residual astigmatism (ORA) after cataract surgery was calculated using preoperative corneal astigmatism, postoperative refractive astigmatism, and surgically induced astigmatism (SIA). Postoperative manifest refraction was measured at least 6 months after surgery and was converted to the corneal plane (Formula 1) by adjusting vertex distance (12 mm), and this was designated as:

Formula 1: Fc = F / (1 – 0.012 × F)

Where Fc is the power corrected for vertex distance and F manifests refractive power.

SIA of temporal clear corneal incision by the surgeon (CYP) was calculated previously as 0.40 diopters.^12^ Therefore, the flattening vector effect of SIA (J0 = 0.20 and J45 = 0). However, SIA is not always same even in single surgeon. To compensate the lack of true SIA calculation using postoperative keratometric data, we applied 2 SIAs (0.20 and 0.40 diopter) to RA and the resulting values were designated as RA1 (SIA of 0.20 diopter) and RA2 (SIA of 0.40 diopter). ORA values were calculated by using RA, RA1, and RA2, respectively.

ORAs were calculated by vector analysis. For example, ORA calculated using AK and RA is as follows:

 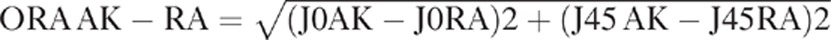


### Subgroup Analysis

Previous study has shown that keratometric astigmatism of IOLMaster overestimates total corneal astigmatism in eyes with the rule (WTR) astigmatism and underestimates it in eyes with against the rule (ATR) astigmatism.^13^ Merging WTR and ATR eyes in single analysis can masquerade the differences. Therefore, the eyes were divided as WTR group when steep axis of astigmatism of AK was measured from 45 to 135 degree and as ATR group when steep axis of astigmatism of AK was measured otherwise.

In further analysis, eyes were divided according to irregularity index (grouping 1 and grouping 2). Grouping 1 designated eyes as low irregular group when irregularity index is <0.036 (median value of 207 eyes) and as high irregular group when irregularity index is ≥0.036. Grouping 2 designated eyes as low irregular group when irregularity index is <0.077 (mean + 2 SD) and as high irregular group when irregularity index is ≥0.077.

### Statistical Analysis

Statistical power was calculated retrospectively based on sample achieved. Sample size of 207 accomplished alpha error probability <0.05 and power (1-beta error probability) of 0.95 in correlation test, student *t* test, Mann Whitney *U* test, paired *t* test, and multiple linear regression analysis when calculated by Gpower: statistical power analysis software (www.gpower.hhu.de).

Statistical analysis was performed using SPSS software ver.20.0 (SPSS Inc, Chicago, IL). Normality of data was assessed by the Shapiro–Wilk test. Student *t* test or Mann Whitney *U* test was used to compare means of different groups. Paired *t* test was used to analyze 2 related variables in the same group. The Pearson correlation coefficient (rho) was used to perform correlation analysis. Multiple linear regression analysis was used to determine the impact of various parameters. *P* values (2-tailed) <0.05 were considered significant.

## RESULTS

The average age of 207 eyes was 68.25 ± 11.33 years (mean ± standard deviation). The clinical characteristics of the eyes are shown in Table 1.

**TABLE 1:**
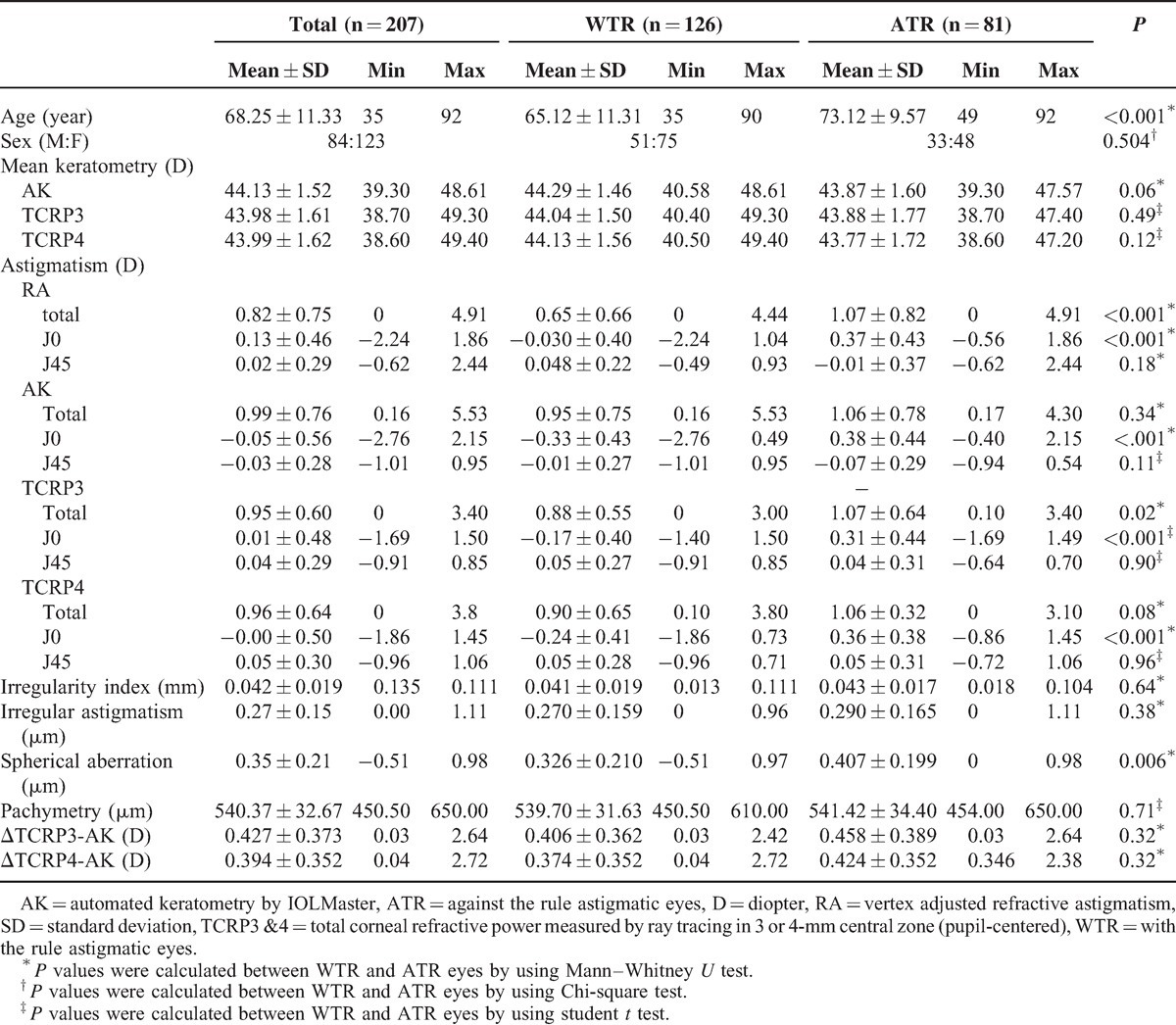
Clinical Characteristics of Total Study Eyes

IR of total study eyes was 0.042 ± 0.019 mm.

**FIGURE 2:**
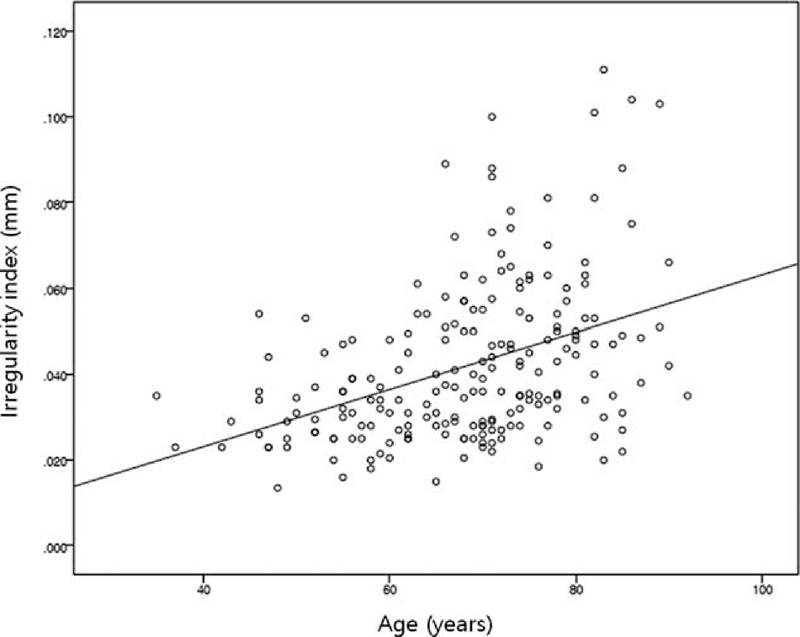
Correlation between age and total corneal irregularity index (IR). Age and IR were positively correlated. Spearman rho = 0.413, *P* < 0.001.

**FIGURE 3:**
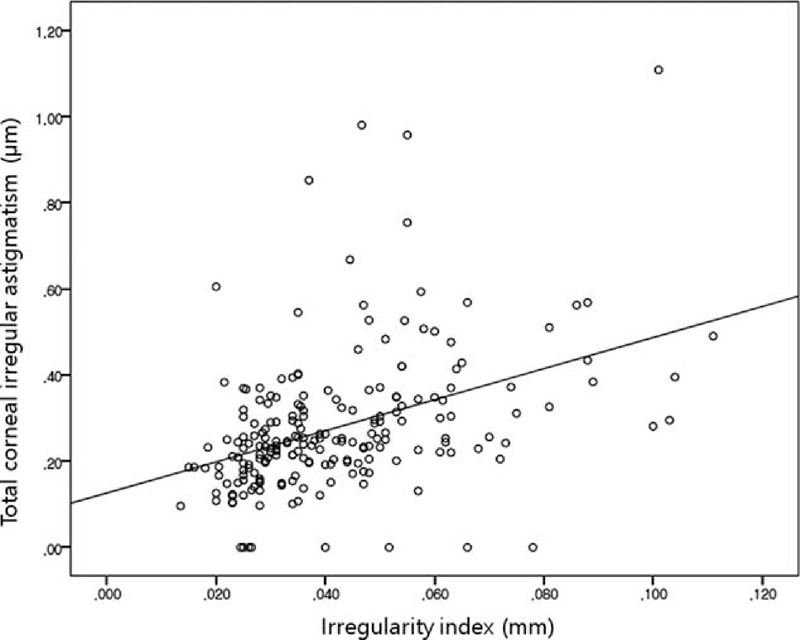
Correlation between corneal irregularity index (IR) and total corneal irregular astigmatism. Spearman rho = 0.473, *P* < 0.001.

Age and total corneal irregular astigmatism correlated significantly with IR (multiple linear regression analysis with logarithmic transformation of IR, *R*^2^ = 0.250; IR = −4.326 + 0.12 × age + 0.683 × total corneal irregular astigmatism) (Figures 2 and 3).

### Difference (Δ) Between 2 Different Strategies of Keratometry

The difference between TCRP and AK (ΔTCRP-AK) was 0.43 ± 0.37 (TCRP3) and 0.39 ± 0.35 (TCRP4) diopters and showed positive correlation with age, IR, and magnitude of astigmatism (AK).

Age, IR, and magnitude of AK correlated significantly with ΔTCRP3-AK (multiple linear regression analysis with logarithmic transformation, *R*^2^ = 0.303; ΔTCRP3-AK =  − 0.388 + 0.007 × age + 1.613 × IR + 0.201 × AK).

Age, IR, and magnitude of AK correlated significantly with ΔTCRP4-AK (multiple linear regression analysis with logarithmic transformation, *R*^2^ = 0.372; ΔTCRP4-AK =  − 0.321 + 0.006 × age + 2.222 × IR + 0.249 × AK).

### Subgroup Analysis with WTR and ATR Astigmatism

The comparison between WTR and ATR group was demonstrated in Table 1. ATR eyes were older (73.12 vs 65.12 years) and had greater magnitude of astigmatism (AK and TCRPs) compared with WTR group. In vector analysis using J0 and J45 component, ATR eyes had more positive J0 component than WTR eyes. And ATR eyes had more corneal spherical aberration (0.407 ± 0.199 μm) than WTR eyes (0.326 ± 0.210 μm). AK and TCRPs measured astigmatism greater than RA, especially in WTR eyes (Tables 1 and 2). This discrepancy was more prominent in the measurement of J0 component rather than J45 component. TCRPs showed no difference in measurement of magnitude of astigmatism compared to AK; however, some significant difference was found when analyzing J0 and J45 component separately. TCRP3 and TCRP4 showed no significant difference each other (Tables 1 and 2). ΔTCRP3-AK and ΔTCRP4-AK showed no difference between WTR and ATR eyes (Table 1).

**TABLE 2:**
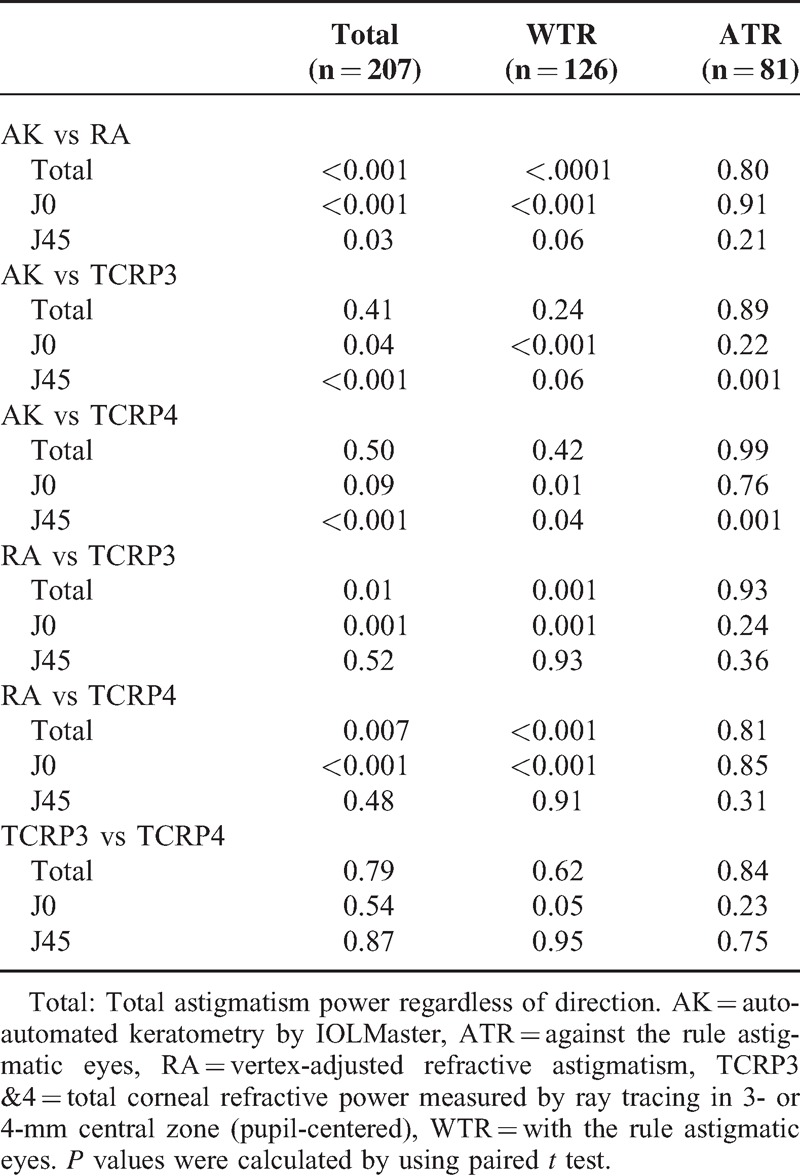
The Comparison of Astigmatism Measurement (Total Power, J0, and J45) by AK and TCRPs in Total, WTR, and ATR eyes. *P* Values Were Demonstrated

### Subgroup Analysis with High and Low Corneal Irregularity

The eyes were divided into 2 groups: high and low IR by 2 different grouping methods described previously. By grouping method 1, eyes in high IR group (n = 97) showed higher irregular astigmatism, age, mean keratometry (TCRPs), and magnitude of astigmatism (RA, AK, and TCRP4). Both ΔTCRP3-AK and ΔTCRP4-AK were higher in high IR group compared with low IR group (n = 110). The anterior to posterior curvature ratio was lower in high IR group (Table 3).

**TABLE 3:**
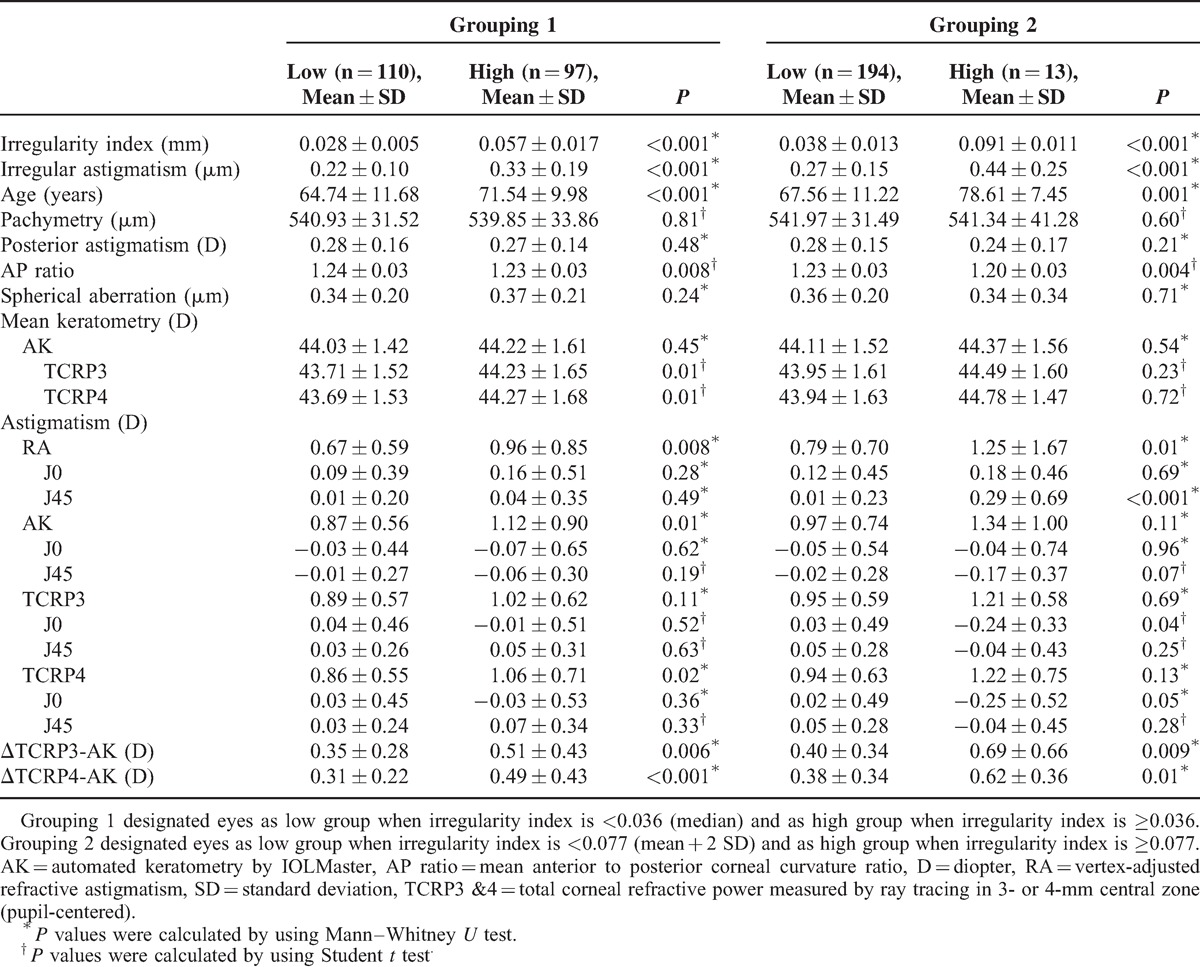
Comparison Corneal Characteristics Between Groups With Low or High Corneal irregularity

Thirteen eyes were included in the high IR group by grouping method 2. Irregular astigmatism and age were higher in the high IR group. However, the magnitude of astigmatism (AK and TCRPs) and mean keratometry values were not different between the high and low IR groups. The magnitude of astigmatism by RA was significantly higher in high IR groups. Although the number of high IR group was small (13 vs 194) in grouping 2, the higher values of both ΔTCRP3-AK and ΔTCRP4-AK were repeatedly verified with statistical significance. The anterior to posterior curvature ratio was lower in high IR group (Table 3).

### ORA Measurements

Because the comparison between WTR and ATR groups showed no difference in ΔTCRP3-AK and ΔTCRP4-AK, ORA comparison was performed only in subgroups divided by IR. As mentioned previously, ORAs were calculated by using RA, RA1, and RA2, respectively, and the results were shown in Table 4. The mean ORAs ranged from 0.43 diopters to 0.51 diopters in 207 eyes.

**TABLE 4:**
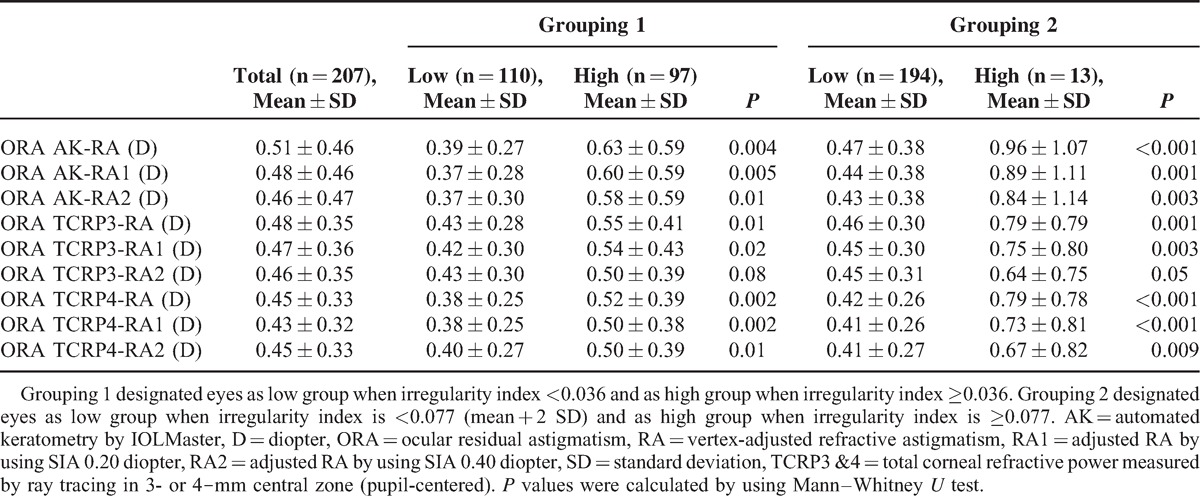
Comparison of ORA Between Groups With Low or High Corneal Irregularity Index

When the eyes were divided into high and low IR groups, ORAs were significantly higher in high IR eyes compared with low IR eyes (Table 4). There was a tendency that ORAs were measured smaller when TCRPs were used rather than when AK was used for the calculation, especially in high IR eyes (Table 4). Statistical analysis revealed that ORAs calculated by using TCRPs were significantly smaller than ORAs calculated by using AK in high IR eyes (Table 5).

**TABLE 5:**
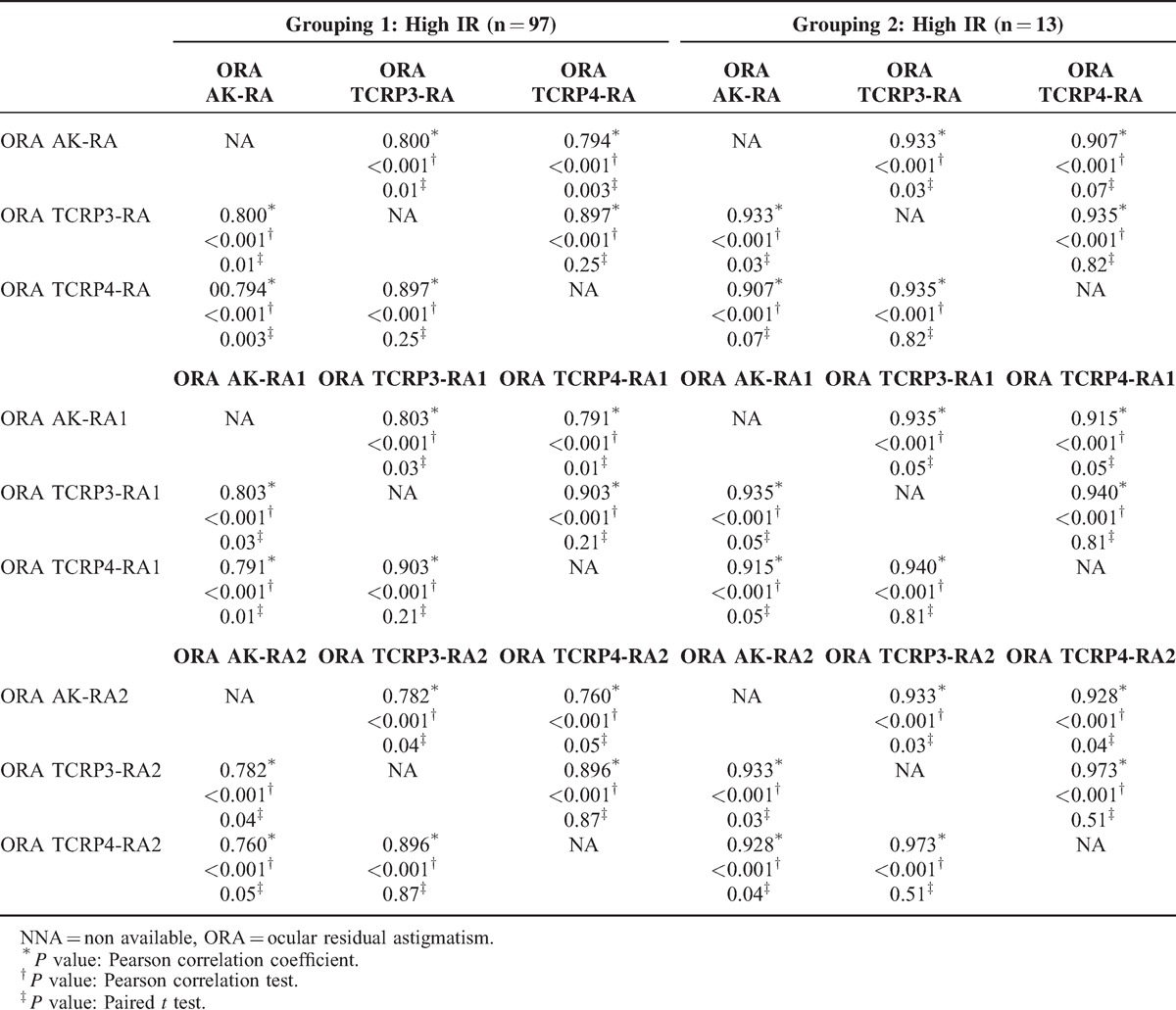
Comparison Between Calculated ORAs Using Different Keratometry in Eyes With High Corneal Irregularity Index. ORAs Calculated by Using AK and RA/RA1/RA2 Were Compared With ORAs Calculated by Using TCRPs and RA/RA1/RA2. Pearson Correlation Coefficient Was Calculated and Paired *t* Test Was Performed

## DISCUSSION

In this study, we found statistically significant differences in corneal astigmatism measurements using 2 different modalities, AK and TCRP, as shown as ΔTCRP-AK. This difference increased as both corneal irregularity and corneal regular astigmatism increased. In addition, by comparing postoperative residual astigmatism, we found that preoperative TCRPs resulted in significantly smaller postoperative ORAs in highly irregular corneas, in other words, a better estimation of postoperative refractive astigmatism.

Corneal astigmatism is very common in patients undergoing cataract surgery.^14–16^ Chen et al^15^ reported that 27.5% of Asians with eyes of cataract age have corneal astigmatism ≥1.25 diopters. De Bernardo et al^16^ reported that 41.74% of eyes of whites have corneal astigmatism ≥1.0 diopters. The residual refractive astigmatism after cataract surgery can significantly decrease a patient's uncorrected visual acuity after cataract surgery.^12^ Toric IOL implantation can neutralize corneal astigmatism and result in minimal ORA.^17^ However, accurate measurement of corneal astigmatism, strict control of SIA, and correct choice of toric IOL power are all prerequisites for successful outcomes.^18^

The importance of posterior corneal astigmatism has been previously emphasized.^17^ Conventional manual or automated keratometers measure only anterior corneal power by using the standard keratometric index of 1.3375. However, using rotating Scheimpflug imaging, independent measurements of both anterior and posterior corneal powers are available. It is believed that the posterior cornea generally compensates for anterior corneal astigmatism and decreases total corneal astigmatism.^19,20^ Therefore, ignoring high posterior corneal astigmatism heightens the risk of astigmatic surprise after a toric intraocular IOL implantation.^18^

The discrepancy between AK and TCRP in this study is expected to some degree. AK using the IOLMaster is measured using six spots of reflection located at the hexagonal apex. The distance of each spot from the visual axis is only about 1.3 mm. Therefore, corneal scars or other focal irregularities can distort the reflective image of dots and may result in an inaccurate measurement of corneal power. Compared with manual keratometry, which measures corneal curvature at 3.0–3.2 mm, AK from the IOLMaster provides more central corneal measurement at about 2.5 mm. Although we analyzed TCRP3 to be closer to 2.5 mm of IOLMaster, still 0.5 mm discrepancy existed. The difference of the reference point used in the measurement, which is the corneal apex in IOLMaster and the pupil center in TCRPs, can be another factor for the discrepancy. Reliability of astigmatism measurement by the IOLMaster is controversial. In a recent study, the agreement between IOLMaster and manual keratometry was evaluated by Bullimore et al.^21^ They reported that the cylinder power measured by the 2 instruments was almost equal and the axis difference was <5 degree in 77.1% of eyes. In another study, corneal astigmatisms measured with the IOLMaster, AK, and Scheimpflug imaging were all comparable to manual keratometry and showed no significant differences.^10^ In contradiction, Shammas et al^22^ reported that the precision of astigmatism measurements by IOLMaster was relatively lower for steeper corneas and the difference in corneal astigmatism measurements between IOLMaster and another automated keratometer (RK-F1, Canon Inc) can increase more in corneas having asymmetric bowtie than in corneas having a symmetric bowtie pattern.^23^ In addition, different studies demonstrate that ignoring posterior corneal astigmatism in AK can result in significant differences both in magnitude and axis of total corneal astigmatism when compared with rotating Scheimpflug imaging.^24,25^ These differences among previous studies may originate from the heterogeneous proportion of irregular corneas included in each study. The agreement between 2 different instruments can be overestimated or underestimated depending on whether a population of highly irregular or moderately irregular corneas is being studied. As shown in our study, a large proportion of high irregular corneas can increase ΔTCRP-AK.

Our finding that Δ TCRPs-AK increased with IR has important clinical significance. This suggests that corneal irregularity should be explored beyond the astigmatism data provided by IOLMaster. In addition, the finding that TCRPs better estimated postoperative refractive astigmatism in highly irregular corneas suggests that measuring both anterior and posterior corneal astigmatism is necessary for surgical planning in these highly irregular corneas. This finding is consistent with previous reports that favor rotating Scheimpflug imaging technology over AK in measuring abnormal corneal shapes.^26–30^ In addition, manual and AK measure corneal powers (sphere and cylinder) with the assumption that the curvature ratio between anterior and posterior cornea is constant.^31,32^ However, in highly irregular corneas, the normal relationship between anterior and posterior corneal curvature is no longer valid. For example, standard keratometry and topography (simulated K values) are often inconsistent when measuring keratoconic corneas.^26,30^ The anterior and posterior curvature ratio was significantly smaller in corneas with high irregularity compared with corneas with low irregularity as shown in our result. Ray tracing technology (TCRP) in rotating Scheimpflug imaging enables corneal power measurement using Snell's law equation for refraction using real refractive index numbers (1 for air, 1.376 for cornea, 1.336 for aqueous). In addition, TCRP zone analysis studies a specific zone and averages the individual corneal powers within that zone. This averaging process may provide permissive effect on corneal irregularity. Previously, TCRPs also showed better performance when compared with AK in measuring corneal power after corneal refractive surgery.^28,29^ We found ORAs calculated using TCRPs resulted in lower ORAs in highly irregular corneas and it means TCRPs are more accurate in astigmatism measurement in irregular corneas. In this study, pupil-centered TCRP was used since postoperative refraction was measured with the pupil centered, and 3 and 4-mm zone was selected to ensure that sufficient corneal area was averaged.

Our study has several limitations. The number of eyes in high IR group by grouping method 2 was only 13. Increasing the number of study eyes for comparison would provide stronger conclusions. However, this subset of 13 eyes with high IR by definition (mean plus 2 standard deviation), among 207 total eyes, is not a small number. Even if the study population is increased, the relative small number in high IR group may not change as long as the strict definition is applied. In some studies, the independent comparison of J0 and J45 was adopted for investigation; however, we used the combined effect of J0 and J45 by using the root of sum of difference square of J0 and J45, and this approach may make the comparison of our results with other studies somewhat difficult. We believe this combined effect has more clinical implications, as surgeons hope for the least amount of residual astigmatism, whether J0 or J45. Another drawback is the lack of postoperative keratometry data and inability to calculate individual SIAs. To minimize this drawback, we applied several different SIAs (0, 0.20, and 0.40 diopters) and calculated RA, RA1, and RA2 values. Separate analyses using different RAs may cover most clinical range of SIAs in temporal clear cornea incisions.

In summary, corneal astigmatism measurements using AK and TCRP were not comparable in corneas with high irregularity. Therefore, careful evaluation of corneal irregularity is necessary before choosing appropriate data for astigmatism correction surgery in these eyes. Significant discrepancy in astigmatism measurement is more common in aged, highly irregular, and more astigmatic corneas. Based on the postoperative ORAs analysis, we conclude that TCRPs are the better predictors of postoperative astigmatism in highly irregular corneas.

## References

[1] Lindsay R Smith G Atchison D Descriptors of corneal shape. *Optom Vis Sci* 1998; 75:156–158. 9503441 10.1097/00006324-199802000-00019

[2] Dave T Current developments in measurement of corneal topography. *Cont Lens Anterior Eye* 1998; 21 Suppl 1:S13–30. 16303392 10.1016/s1367-0484(98)80034-9

[3] Oliveira CM Ribeiro C Franco S Corneal imaging with slit-scanning and Scheimpflug imaging techniques. *Clin Exp Optom* 2011; 94:33–42. 20718786 10.1111/j.1444-0938.2010.00509.x

[4] Aptel F Chiquet C Gimbert A Anterior Segment Biometry Using Spectral-Domain Optical Coherence Tomography. *J Refract Surg* 2014; 30:354–360. 24694582 10.3928/1081597X-20140326-01

[5] Karnowski K Kaluzny BJ Szkulmowski M Corneal topography with high-speed swept source OCT in clinical examination. *Biomed Opt Express* 2011; 2:2709–2720. 21991558 10.1364/BOE.2.002709 PMC3184879

[6] Nakagawa T Maeda N Higashiura R Corneal topographic analysis in patients with keratoconus using 3-dimensional anterior segment optical coherence tomography. *J Cataract Refract Surg* 2011; 37:1871–1878. 21930048 10.1016/j.jcrs.2011.05.027

[7] Higashiura R Maeda N Nakagawa T Corneal topographic analysis by 3-dimensional anterior segment optical coherence tomography after endothelial keratoplasty. *Invest Ophthalmol Vis Sci* 2012; 53:3286–3295. 22628206 10.1167/iovs.12-9602

[8] Crawford AZ Patel DV McGhee CN Comparison and repeatability of keratometric and corneal power measurements obtained by Orbscan II, Pentacam, and Galilei corneal tomography systems. *Am J Ophthalmol* 2013; 156:53–60. 23540708 10.1016/j.ajo.2013.01.029

[9] Huang J Ding X Savini G A Comparison between Scheimpflug imaging and optical coherence tomography in measuring corneal thickness. *Ophthalmology* 2013; 120:1951–1958. 23672973 10.1016/j.ophtha.2013.02.022

[10] Lee H Chung JL Kim EK Univariate and bivariate polar value analysis of corneal astigmatism measurements obtained with 6 instruments. *J Cataract Refract Surg* 2012; 38:1608–1615. 22795977 10.1016/j.jcrs.2012.04.035

[11] Goto T Klyce SD Zheng X Gender- and age-related differences in corneal topography. *Cornea* 2001; 20:270–276. 11322415 10.1097/00003226-200104000-00007

[12] Park CY Chuck RS Residual refractive error and visual outcome after cataract surgery using spherical versus Aspheric IOLs. *Ophthalmic Surg Lasers Imaging* 2011; 42:37–43. 21117581 10.3928/15428877-20101124-04

[13] Koch DD Jenkins RB Weikert MP Correcting astigmatism with toric intraocular lenses: effect of posterior corneal astigmatism. *J Cataract Refract Surg* 2013; 39:1803–1809. 24169231 10.1016/j.jcrs.2013.06.027

[14] Hoffmann PC Hutz WW Analysis of biometry and prevalence data for corneal astigmatism in 23 239 eyes. *J Cataract Refract Surg* 2010; 36:1479–1485. 20692558 10.1016/j.jcrs.2010.02.025

[15] Chen W Zuo C Chen C Prevalence of corneal astigmatism before cataract surgery in Chinese patients. *J Cataract Refract Surg* 2013; 39:188–192. 23141077 10.1016/j.jcrs.2012.08.060

[16] De Bernardo M Zeppa L Cennamo M Prevalence of corneal astigmatism before cataract surgery in Caucasian patients. *Eur J Ophthalmol* 2014; 24:494–500. 24366768 10.5301/ejo.5000415

[17] Savini G Hoffer KJ Ducoli P A new slant on toric intraocular lens power calculation. *J Refract Surg* 2013; 29:348–354. 23659233 10.3928/1081597X-20130415-06

[18] Alpins N Ong JK Stamatelatos G Refractive surprise after toric intraocular lens implantation: graph analysis. *J Cataract Refract Surg* 2014; 40:283–294. 24461500 10.1016/j.jcrs.2013.06.029

[19] Park CY Oh JH Chuck RS Predicting ocular residual astigmatism using corneal and refractive parameters: a myopic eye study. *Curr Eye Res* 2013; 38:851–861. 23621376 10.3109/02713683.2013.790976

[20] Hashemi H Khabazkhoob M Peyman A The association between residual astigmatism and refractive errors in a population-based study. *J Refract Surg* 2013; 29:624–628. 23799793 10.3928/1081597X-20130620-01

[21] Bullimore MA Buehren T Bissmann W Agreement between a partial coherence interferometer and 2 manual keratometers. *J Cataract Refract Surg* 2013; 39:1550–1560. 23876813 10.1016/j.jcrs.2013.03.034

[22] Shammas HJ Chan S Precision of biometry, keratometry, and refractive measurements with a partial coherence interferometry-keratometry device. *J Cataract Refract Surg* 2010; 36:1474–1478. 20692557 10.1016/j.jcrs.2010.02.027

[23] Park JH Kang SY Kim HM Differences in corneal astigmatism between partial coherence interferometry biometry and automated keratometry and relation to topographic pattern. *J Cataract Refract Surg* 2011; 37:1694–1698. 21855766 10.1016/j.jcrs.2011.03.047

[24] Ho JD Tsai CY Liou SW Accuracy of corneal astigmatism estimation by neglecting the posterior corneal surface measurement. *Am J Ophthalmol* 2009; 147:788–795.95 e1-2. 19232562 10.1016/j.ajo.2008.12.020

[25] Koch DD Ali SF Weikert MP Contribution of posterior corneal astigmatism to total corneal astigmatism. *J Cataract Refract Surg* 2012; 38:2080–2087. 23069271 10.1016/j.jcrs.2012.08.036

[26] Thebpatiphat N Hammersmith KM Rapuano CJ Cataract surgery in keratoconus. *Eye Contact Lens* 2007; 33:244–246. 17873627 10.1097/ICL.0b013e318030c96d

[27] Symes RJ Say MJ Ursell PG Scheimpflug keratometry versus conventional automated keratometry in routine cataract surgery. *J Cataract Refract Surg* 2010; 36:1107–1114. 20610087 10.1016/j.jcrs.2009.11.026

[28] Oh JH Kim SH Chuck RS Evaluation of the Pentacam ray tracing method for the measurement of central corneal power after myopic photorefractive keratectomy. *Cornea* 2014; 33:261–265. 24322808 10.1097/ICO.0000000000000034

[29] Savini G Hoffer KJ Carbonelli M Scheimpflug analysis of corneal power changes after myopic excimer laser surgery. *J Cataract Refract Surg* 2013; 39:605–610. 23465330 10.1016/j.jcrs.2012.12.031

[30] Watson MP Anand S Bhogal M Cataract surgery outcome in eyes with keratoconus. *Br J Ophthalmol* 2014; 98:361–364. 23966369 10.1136/bjophthalmol-2013-303829

[31] Seitz B Langenbucher A Intraocular lens calculations status after corneal refractive surgery. *Curr Opin Ophthalmol* 2000; 11:35–46. 10724826 10.1097/00055735-200002000-00006

[32] Seitz B Langenbucher A Intraocular lens power calculation in eyes after corneal refractive surgery. *J Refract Surg* 2000; 16:349–361. 10832985 10.3928/1081-597X-20000501-09

